# Reinforcement Learning-Based Intelligent Path Planning for Optimal Navigation in Dynamic Environments

**DOI:** 10.1007/s11063-025-11821-2

**Published:** 2026-01-04

**Authors:** Anil Kumar Yadav, Purushottam Sharma, Xiaochun Cheng, Shiv Shankar Prasad Shukla

**Affiliations:** 1 VIT Bhopal University, Bhopal-Indore Highway, Bhopal, India; 2 School of Computer Science and Engineering, Galgotias University, Greater Noida, India; 3 Computer Science Department, Bay Campus Fabian Way, Swansea University, Swansea, SA1 8EN Wales UK

**Keywords:** Q-learning (QL), Reinforcement learning (RL), Reward function, Policy iteration, Path optimization, Trajectory planning, Navigation

## Abstract

Path selection and planning are crucial for autonomous mobile robots (AMRs) to navigate efficiently and avoid obstacles. Traditional methods rely on analytical search to identify the shortest distance. However, Reinforcement learning enhances performance by optimizing a sequence of actions efficiently. It is an iterative approach used for computational sequence modeling and dynamic programming. RL received sensory input from the environment in the form of observation or state. The agent interpreted every reward or penalty through trial-and-error interaction. Policy maximizes the rewards and selects the optimal action among all possible actions. A challenging problem in traditional reinforcement learning is environment generalization for dynamic systems. Q-learning faces challenges in dynamic environments because it relies on rewards or penalties based on the entire sequence of actions from the start to the end state. This approach often fails to produce optimal results when the environment changes unexpectedly due to state transitions, iterations, or blocked routes. Such limitations make Q-learning less effective for dynamic path planning. To overcome these challenges, this study focuses on optimizing reward functions for efficient navigation in RL-based path planning, aiming to enhance navigation efficiency and obstacle avoidance. The proposed method evaluates the shortest decision path by considering total steps, counted steps, and discount rates in dynamic environments. By implementing this RL with an optimized reward mechanism, the study analyzes state reward values across different environments, and it evaluates the effect on state-action pair-based Q-Learning and neural networks using Deep Q-Learning algorithms. Here, results demonstrate that the optimized reward function effectively decreases the number of iterations and episodes while achieving a 30% to 70% reduction in overall trajectory distance. These results highlight the effectiveness of reward-based reinforcement learning, demonstrating its potential to improve path optimization, learning rate, episode completion, and decision accuracy in intelligent navigation systems. Q-learning-based reinforcement learning becomes more effective by combining multiple agents and utilizing decision-making techniques such as federated and transfer learning on larger maps to ensure convergence.

## Introduction

Path planning has become a crucial area of research in autonomous mobile robotics, aiming to minimize time and energy consumption. Efficient path planning is a significant challenge in robotic systems, particularly in terms of motion control and environmental awareness. The primary goal is to develop analytical solutions that enable agents to identify optimal trajectories with minimal distance [1]. Various methods have been designed to allow robots to guide the agent from the start point to the target, while accounting for predefined object coordinates [2]. When selecting a decision path, an agent must navigate around all types of obstacles. In the current scenario, incorporate environmental variables to enhance path planning efficiency [3–5]. Path searching and planning have become essential components of navigation systems. While many algorithms prioritize finding the shortest path, other factors, such as the search area and time complexity, are also considered for optimal performance. Cell decomposition is a technique used in route planning that divides the environment into smaller regions. Graph algorithms help find the shortest path with minimal delay. Dijkstra’s and A* algorithms ensure effective path planning based on the current and given endpoints [6]. Another commonly used method is edge connection, which ensures that no overlap occurs between the lines of objects and describes a repulsive force around obstacles to aid in navigation [7, 8]. Expanding a search tree from the starting point toward the goal until a valid path is established is described as a rapidly exploring random tree (RRT) [9]. Many intelligent systems integrate advanced ANN and GA, with choice techniques depending on recent environmental information [10, 11]. Global and local path planning can incorporate innovative methods such as cell decomposition, which discretizes vacant space. Mathematical graphs offer high accuracy and are effectively utilized in conjunction with automated reasoning and artificial intelligence, particularly when considering multiple variables [12].

Reinforcement learning addresses key challenges in sequential decision-making, including control problems in gaming, computational modeling, and various applications in machine learning, operations research, power grid distribution, and control engineering [13–15]. Several studies have demonstrated that reinforcement learning algorithms can effectively estimate the shortest distance from the current state to the endpoint in both static and dynamic environments [16–18].

Reinforcement Learning (RL) is a method of training agents to learn optimal decision-making by mapping situations to actions in a way that maximizes a numerical reward signal. In RL, the agent interacts with an environment, taking actions and receiving rewards or penalties based on its choices. Through repeated movement, it gradually learns the best policy, allowing it to navigate and adapt dynamically to achieve its objective. However, reinforcement learning has certain drawbacks, such as the need for a large lookup table to store state-action pairs in extensive environments, resulting in high memory requirements due to numerous iterations. Additionally, increasing the grid size exponentially can slow down the convergence rate [19–21].

This study aims to enhance algorithm performance by updating the reward function to determine the shortest path trajectory in robotics. To achieve this, an innovative approach is introduced to modify the reward function, reducing the iterative process and increasing the discount rate during navigation in both static and dynamic environments [22, 23]. This approach is evaluated against machine learning techniques, such as Q-learning algorithms, Deep Q-learning, and Temporal Difference Learning. In some cases, state-action pair tables are modified using a dynamic environment, along with the stuck state. By initializing the temporary memory Q (s, a) effectively, the learning process is accelerated [24]. A dynamic lookup table, where Q-values are adjusted based on the distance between obstacles, enhances the Q-Learning algorithm and delay time. Additionally, this approach increases the reward for each state in path planning algorithms [25, 26]. The smoothing trajectory and diverse trajectory both yield satisfactory outcomes, as demonstrated by Genetic Algorithms [27].

The main focuses of this study are as follows:

- The proposed modified reward function has demonstrated high accuracy and outperformed other models in comparison.
- The optimized reward function efficiently evaluates the shortest distance and time trajectories for prey capture while reducing the number of episodes.
- A supervised NN classifier is employed to find the shortest decision path based on the proposed method.
- A comparative analysis of proposed algorithms with various RL algorithms
- The implementation is tested using Deep Q-Learning (DQL) and the Double Deep Q Network (DDQN), with training conducted based on the grid world problem.
- The key novelty of the proposed optimized reward function is that it dynamically balances path optimality, obstacle avoidance, and smooth trajectory generation in changing environments. While traditional Q-Learning and Deep Q-Learning reward formulations primarily consider distance or collision penalties, our optimized reward integrates a progressive distance reward, a dynamic obstacle penalty, and path smoothness.

To assess algorithm efficiency in the context of discount rate, execution time, and memory usage, it is compared with three algorithms: Deep Q-learning (DQL), Double Deep Q Network (DDQN), and Temporal Difference Learning (TDL).

The summary of this paper is as follows: Sect. 2 reviews relevant studies on navigation planning, while Sect. 3 outlines the proposed methodology, including algorithms and the Deep Q-Learning model. Section 4 presents the experimental results, and Sect. 5 concludes the study with recommendations for future research.

## Related Work

Model-free learning is a type of reinforcement learning that is commonly applied in areas such as autonomous driving, intelligent control systems, and brain research. It provides practical solutions for decision-making in unknown environments and operates as a model-free learning system based on trial-and-error interactions. Q-learning evaluates the quality of actions through an evaluation function to determine the shortest trajectories for robotic navigation.

The Markov Decision Process (MDP) is a mathematical model used to solve decision-making problems and describe how an agent behaves in a given environment. Q-Learning, a fundamental RL algorithm, begins without prior knowledge; it aims to discover the shortest-path solutions. By leveraging MDP, an agent executes a sequence of actions to optimize performance, storing the value of every action pair in the Q matrix. In problems with multiple states, selecting the optimal action for each state is crucial to achieving a higher reward. Policy iteration, a key reinforcement learning technique, involves various executions of the repeated process, refining the agent’s decision-making capabilities. Rapidly exploring the random tree concept of navigation and path planning is closely associated with automated driving systems and vision-based mapping techniques. These methods are utilized to assess traffic congestion and enhance the accuracy of trajectory estimation with minimal distance. The RRT* algorithm is effective in identifying the shortest route while minimizing overall cost [28]. Trajectory tracking is a crucial control problem that utilizes the Control Lyapunov Function (CLF) to determine the minimum distance between two points. It is applied to finding the trajectory of a two-wheeled mobile robot along with time-varying goal states [29].

This study presents a mechanism for determining an obstacle-free optimal path for mobile robots operating in a radioactive environment [30]. The Deep Double Q-Network (DDQN) is an efficient approach for finding trajectory paths and planning. Additionally, an action selection method is used for supervised artificial networks. That is utilized to determine action values for the given environment. For each Iteration, the agent’s reward is used to assess the algorithm’s efficiency in complex grid environments [31, 32]. One challenge in reinforcement learning is creating a practical reward function that effectively guides the agent. Establishing new reward functions enhances optimal navigation by enabling autonomous mobile robots (AMR) to find the shortest trajectory with minimal distance [11]. The Double Deep Q-Network (DDQN) is an enhanced reinforcement learning algorithm designed to overcome the overestimation bias inherent in the traditional Deep Q-Learning (DQN) approach. In DQN, the same neural network is used both to select and evaluate actions, which often leads to inflated value estimates and unstable learning. To address this issue, DDQN decouples the action selection and evaluation processes by employing two separate networks. The online network is responsible for choosing the best possible action, while the target network evaluates the expected return for that action. This separation leads to more stable convergence and improved decision accuracy, particularly in dynamic or continuous environments [33].

Comprehensive analysis of path planning methodologies, encompassing classical, metaheuristic, and artificial intelligence (AI)-driven approaches. Metaheuristic techniques, including Genetic Algorithms (GA), Particle Swarm Optimization (PSO), and Ant Colony Optimization (ACO), offer enhanced adaptability and global search capabilities; however, they may suffer from slower convergence and increased computational costs in large-scale or real-time applications [34]. Traditional path planning and obstacle avoidance techniques often struggle to ensure safe and efficient flight in these cluttered, height-restricted environments. Intelligent navigation methods that leverage advanced sensing, perception, and learning capabilities. Soft Actor-Critic (SAC)-based approaches have demonstrated promise in developing robust navigation policies that can handle real-time obstacle detection and avoidance while maintaining flight stability within constrained airspaces. These learning-based frameworks enhance situational awareness and decision-making by continuously updating the UAV’s navigation strategy based on environmental feedback [35]. Ensuring the safe operation of Robotic Autonomous Systems (RAS) within highly regulated environments has become a critical research focus, especially as these systems are increasingly deployed in sectors such as healthcare, manufacturing, and transportation. Integrating this rule-based safety layer with the robot’s autonomy module, RAS can make intelligent decisions while maintaining strict adherence to safety protocols [36].

RL methods act through trial-and-error interactions between the agent and the environment. A well-designed reward function encourages the decision-making system to take the shortest trajectory, while minimizing the number of iterations required for convergence.

## Proposed Methodology

An optimized reward function is used to update the state-action pair of robots over multiple iterations. The robot navigation problem is addressed through environment modelling, trajectory planning algorithms, and a schematic workflow of Deep Q-learning. Environment modelling is represented using the cell decomposition method, where the space is divided into equal-sized cells while accounting for both static and random Stuks. Introduced path planning incorporates tabular and artificial neural network-based techniques. Both approaches aim to guide the agent toward selecting the shortest routes with fewer iterations. Additionally, the schematic workflow diagram outlines the training procedure for RL-based Q-Learning and DQL, illustrating a step-by-step process.

### Environment Modelling

**Fig. 1:**
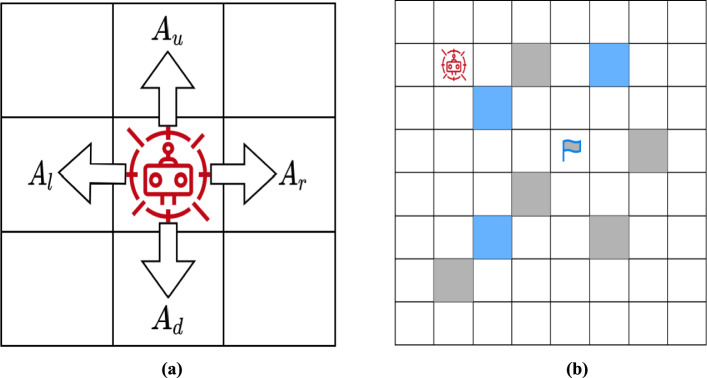
The interaction between various states and corresponding actions within the environment. **a** An agent is permitted to take four possible actions: left, right, up, and down. **b** Cell decomposition representation of the environment at two different time instances, where grey obstacles indicate static route blockages and blue obstacles represent dynamic route blockages

The environment is constructed using the cell decomposition technique, which transforms the problem into a finite-state representation for designing RL algorithms. Each cell represents a specific position, movement, and possible states relevant to the problem, making it easier to address navigation challenges. Within this environment, the robot is modeled as an agent, represented by a small circle smaller than the cell size. The agent is allowed to move in four directions—left, right, up, and down—while diagonal movements are restricted, as illustrated in Fig. 1.

The environment consists of an 8 × 8 grid of cells, where obstacle positions vary dynamically. Figure 1b illustrates an example of the environment, where grey and blue-filled cells indicate static and dynamic obstacles, respectively. The target point is marked with a flag, while the robot is initially positioned at coordinates (2, 2) with a designated target at (4, 5). Each cell within the grid represents a distinct (x, y) coordinate, where x and y are integer values.

**Fig. 2:**
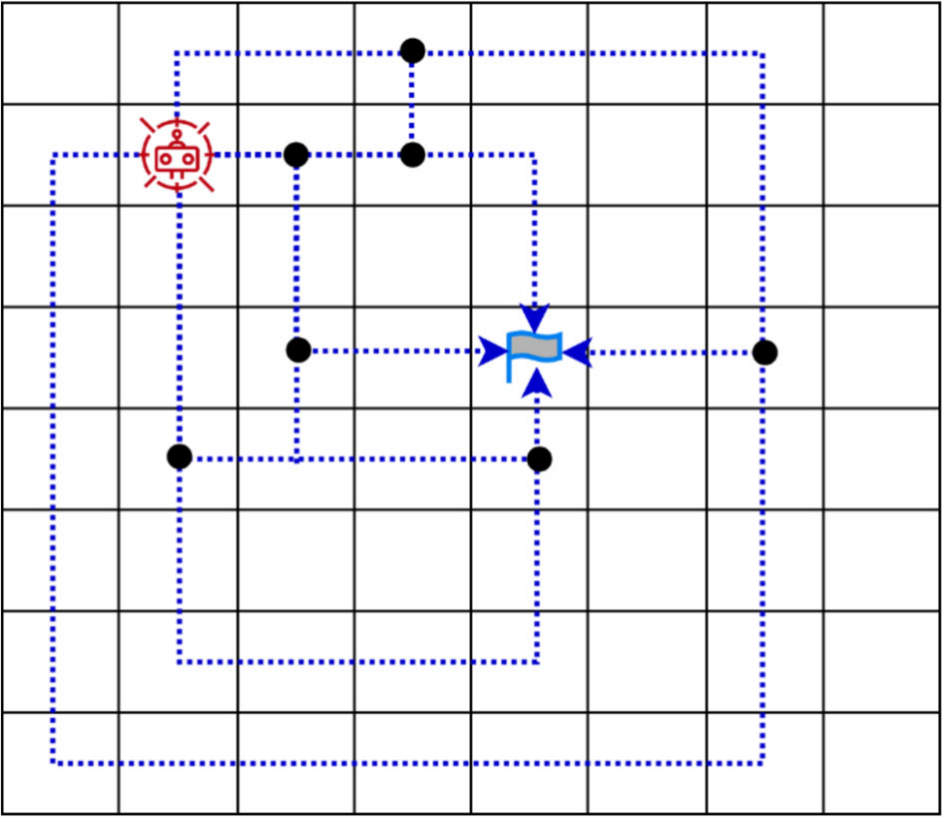
Possible routes for the robot towards the goal

In Fig. 2, finding a fixed prey (or goal) in a dynamic environment is illustrated using reinforcement learning. The prey remains stationary at a fixed position, while both static and dynamic obstacles obstruct the path from the predator (hunter) to the prey. The objective is to capture the prey in the shortest possible steps while avoiding these obstacles. The predator can move one step in any of the potential directions up, down, left, or right, with diagonal movements restricted.

### Reinforcement Learning

**Fig. 3:**
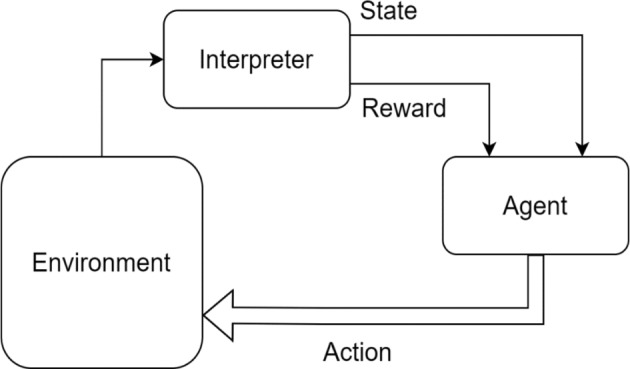
The agent–environment interaction in Reinforcement learning

Figure 3 illustrates the interaction between the agent and the environment, where the environment—supported by an interpreter—simulates a real-world scenario by evaluating the agent’s actions and returning feedback in the form of rewards or penalties, along with an updated state. The agent generates actions in response to receiving input from the environment, which is presented in the form of states or observations. It utilized the Marko decision process (MDP) to select the best action among the available options. The agent learns in many iterations under a defined action selection policy that is responsible for modifying decisions and selecting the best action over a given environment. Self-critics, also known as interpreters, play a role in determining the best course of action during movement. Reinforcement learning is a powerful tool for solving problems without prior knowledge of the system.

Each time step *t* occurs, the agent observes an abstract piece of information (which we call a state) and a numerical quantity (which we call reward in absolute numbers).

A random state and reward are served towards the possible movement *t* + 1 state and action, respectively. The environment provides several observations along with a random variable:

- State/Reward progression is solely dependent on the current state of a given action.
- A complete reward is determined through the agent’s trial-and-error interactions with the environment. It is sufficient to examine the existing situation to predict the future consequences of a given action.

The policy function transforms a state into an action at each given time step. Hence, the agent’s role involves exercising control by determining the best policy function—innovative contribution—to develop an efficient and rewarding approach to a reinforcement Learning algorithm for a dynamic environment.

State actions sequence defined as a set of states s_t,_ action a_t_, and reward R_t_, every moment per step t = 0, 1, 2, 3, … The initial sequence can be represented as Eq. (1)

1 $$ S_{0} ,A_{0} ,R_{1} ,S_{1} ,A_{1} ,R_{2} \ldots $$

Maximizing the reward in terms of performance R, which represents commutative rewards, can be expressed in Eq. (2).

2 $$ R = R_{t + 1} + \, R_{t + 2} + \, R_{t + 3} \cdots + R_{t} $$

Equation (3) defines the reward terms for this study, which aim to achieve two key objectives:

(i) Enhancing the agent’s potential movements and (ii) reducing the frequency of direction changes.

3 $$ Q\left( {s, a} \right) = r \left\{ {\begin{array}{*{20}l} {\left( {1 - \alpha } \right)Qt\left( {st, at} \right) + \alpha t\left[ {rt + \gamma \max Q\left( {st + 1,a} \right)} \right]} \hfill & {} \hfill \\ {rt + \gamma \max Q\left( {st + 1,at + 1} \right),} \hfill & {{\mathrm{if}}\, \alpha t = 1} \hfill \\ {Q\left( {st,at} \right)} \hfill & {{\mathrm{if}}\,\alpha t = 0} \hfill \\ \end{array} } \right. $$

The discount rate γ and is denoted as $\gamma \in \left( {0, \, 1} \right)$. The returned reward values r_t_ are obtained from the environment, and α represents the agent’s learning rate [13].

However, the reward in each episode may vary. In this context, the maximization of the expected reward is expressed in Eq. (4).

In this context, the expected reward maximization is represented by Eq. (4):

4 $$ R = \mathop \sum \limits_{i = 0}^{\infty } \gamma^{t} r_{t + 1} , \ldots ,\quad 0 < \gamma < 1, $$

where the discount rate, $\gamma \in \left( {0,1} \right)$ and r_t_ represent the reward values returned from the environment [11].

The proposed optimized reward, derived from Eq. (4), is represented by the mathematical expression in Eq. (5) using rewards and discount rates.

5 $$ {\mathrm{Optimized}}\,{\mathrm{Reward}}\,R_{1} = \mathop \sum \limits_{i = 0}^{\infty } \gamma^{{\left( {x - i} \right)}} r_{t} $$

The derivation starts from traditional Q learning Q (s, a) ← Q (s, a) $\alpha [r + \gamma \max Q(s^{\prime } ,a^{\prime } ) - Q \, (s, \, a)]$, optimized reward function r as a weighted combination of the three components defined as $r = m_{1} r_{d} + m_{2} r_{o} + m_{3} r_{g}$, where r_d,_ r_o,_ r_g_ correspond to distance efficiency, obstacle interaction, goal alignment rewards, respectively. The weight parameters m_1_, m_2,_ m_3_ are adaptively tuned using feedback from environmental dynamics to maintain a balanced learning process.

The reward values r_t_ are obtained from the environment, where x represents the total number of steps between the start and end, and I is the current step.

### RL-Based Q Learning Algorithm

Reinforcement learning encompasses three key approaches: State-Action-Reward-State-Action (SARSA) [37], Q-Learning [38], and Markov Decision Process (MDP). SARSA is a decision-making system that stores state-action values in a lookup table. These methods are used to assess the value of each state-action pair, guaranteeing convergence regardless of the policy followed by the agent [13]. The lookup table, representing a Quality Matrix of dimensions N × Z, stores state-action data during the modeling of action sequences. Here, N denotes the number of possible states (observations) used to perceive the environment, while Z represents the set of possible actions available to the agent. Q-learning functions in a discrete state-action (SA) space, where the best possible action is chosen by identifying the maximum reward value from the reward matrix. At the start of the training phase, the Q-Matrix is initialized with values set to either random numbers or zero, and it is subsequently updated using Q-learning, as described in Eq. (6).

6 $$ R_{1} = R\gamma^{{\left( {x - i} \right)}} $$

Here, **R** represents the total accumulated reward, **R1** denotes the reward value for the current state, **x** is the total number of steps, and **i** indicates the current step count.

**Fig. 4:**
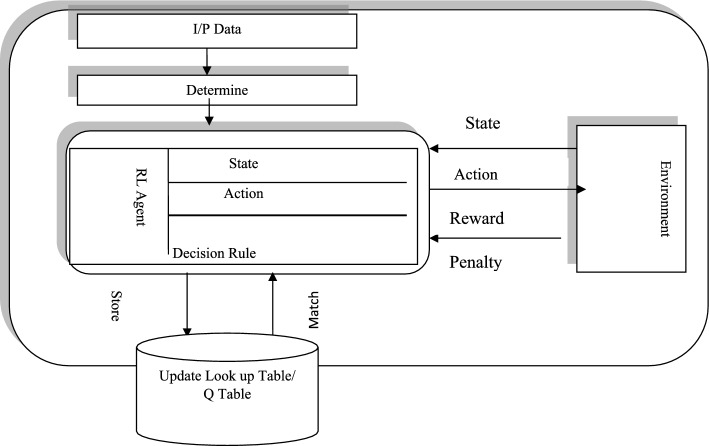
Reinforcement learning framework using Q-learning strategy

It determines the extent to which the agent should prioritize long-term rewards over immediate gains (Fig. 4).

**Algorithm 1:**
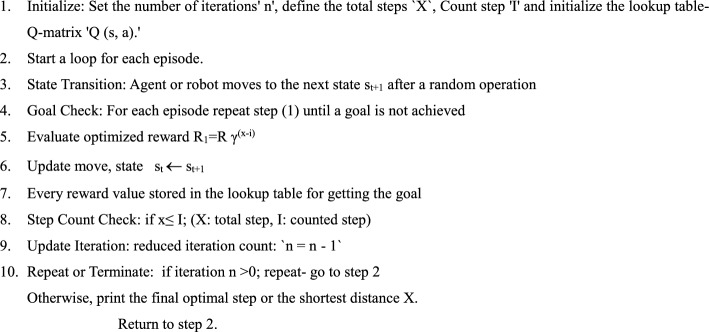
Q learning

Table 1 outlines the training parameters used to assess the optimal discount rate in Q-Learning, guiding the decision-making agent to prioritize maximizing cumulative rewards. Conversely, a lower convergence is adopted to ensure convergence, which increases the required repetition. For experimental analysis, iteration reduction is employed to distinguish between QL and DQL techniques.

**Table 1:** Overview of Q-learning model parameter training procedures

Feature	Range
Episode	10–60
Rate of discount	0.99
Iteration	1–500
Learning rate	0.001

The algorithm was developed and executed in MATLAB on a system running Windows 11. Reinforcement learning is a model-free technique that relies on state-action-reward pairs and policy updates through a Q-matrix. Each row in the Q-matrix represents state-action values, where Q denotes the quality of each action executed by the agent within a grid or defined space. The reward matrix is automatically updated in the lookup table using the Bellman equation, enabling the selection of the best action for each state.

**Fig. 5:**
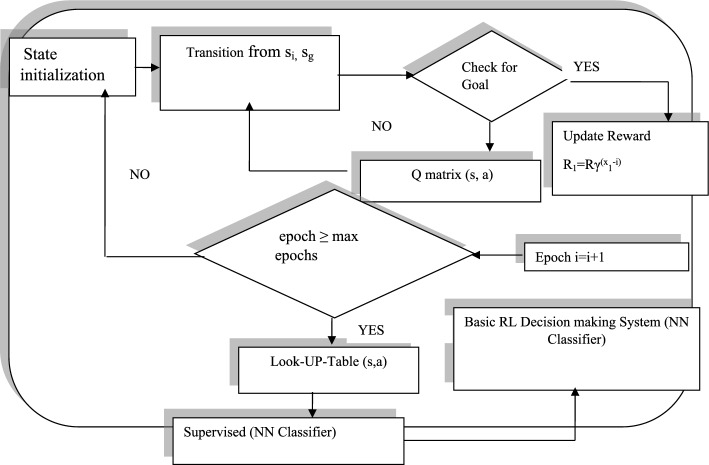
Schematic diagram of reinforcement learning model with supervised neural network integration for deep Q-learning

Figure 5 shows the workflow of supervised learning combined with an artificial neural network classifier based on Deep Q-Learning (DQL), where the state-action pair Q(S, a) is updated using the immediate reward received. The training process is terminated when the number of epochs exceeds the predefined maximum limit (epoch ≥ max epochs) or when the change in Q-values between successive iterations becomes smaller than a convergence tolerance.

The deep Q-learning algorithm leverages a supervised neural network to identify the optimal action from all possible actions in both dynamic and static environments. The robot aims to perform the most effective action by maximizing the reward through the shortest decision path. Algorithm 2 outlines the training and testing procedure for Deep Q-Learning, enabling an agent or robot to operate effectively in a dynamic environment. The agent selects a random action with probability ε to encourage exploration of unvisited states and the action with the highest Q-value with probability (${1}{-}\upvarepsilon$) to promote exploitation of learned knowledge. The value of ε gradually decayed as training progressed to ensure convergence toward an optimal policy. In Deep Q-Learning, the exploration rate (ε) determines how the agent balances exploratory behaviour with the use of its current best actions. When ε is high, the agent performs more random actions to discover new state–action combinations. As training advances, ε is systematically decreased, allowing the agent to rely more on the learned Q-values for informed decision-making. Replay memory stores previous transitions, including state, action, and reward, and plays a crucial role in stabilizing neural network updates in DQN. By maintaining a sufficiently large memory buffer, the algorithm can sample diverse past experiences, reducing the correlation between consecutive observations and promoting more stable learning. This mechanism improves convergence behaviour, enhances training stability, and contributes to more efficient learning over time.

**Algorithm 2:**
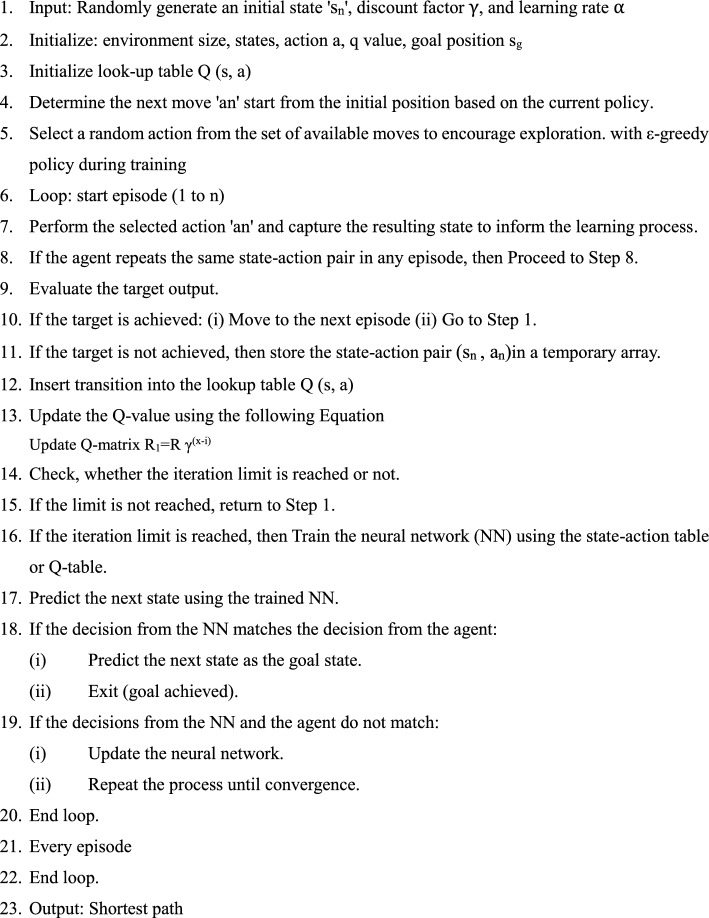
Deep Q-learning to navigate and adapt effectively within continuously changing environments

Table 2 outlines the training parameters applied in this study. A higher discount factor encourages the decision-making agent to prioritize long-term rewards, while a lower learning rate leads to slower convergence, requiring a greater number of training episodes.

**Table 2:** Lists the training parameters used for implementing the deep Q-learning (DQL) techniques

Features	Range
Probability distribution	1
Rate of discount	0.99
Size of step parameter	1
Probability of random action	0.1
Possible agent action	4
Learning rate, *α*	0.001
Number of episodes	12,000

Reinforcement learning techniques were implemented using MATLAB, and the same hardware setup as the Q-learning algorithm was used.

### Look-Up-Table

The state-action pair, also referred to as the Q-matrix or lookup table, stores state-action values in the form of state-action pairs, as shown in Table 3.

**Table 3:** Reward values corresponding to the grid world environment depicted in Fig. 6

Actions	States				Total reward	Ways
	$A_{u}$	$A_{d}$	$A_{l}$	$A_{r}$		
$S_{11}$	N/A	0.32	N/A	0.32	0.64	2
$S_{12}$	N/A	0.42	0.24	0.42	1.08	3
$S_{13}$	N/A	0.56	0.32	0.56	1.44	3
$S_{14}$	N/A	0.75	0.42	N/A	1.17	2
$S_{21}$	0.24	0.42	N/A	0.42	1.08	3
$S_{22}$	0.32	0.56	0.32	0.56	1.76	4
$S_{23}$	0.42	0.75	0.42	0.75	2.34	4
$S_{24}$	0.32	1.0	0.56	N/A	1.88	3
$S_{31}$	0.32	0.32	N/A	0.56	1.20	3
$S_{32}$	0.42	0.42	0.24	0.75	1.83	4
$S_{33}$	0.56	0.56	0.32	1.0	2.44	4
$S_{34}$	0	0	0	0	0	0
$S_{41}$	0.42	N/A	N/A	0.42	0.84	2
$S_{42}$	0.56	N/A	0.32	0.56	1.44	3
$S_{43}$	0.75	N/A	0.42	0.75	1.92	3
$S_{44}$	1.0	N/A	0.56	N/A	1.56	2

**Fig. 6:**
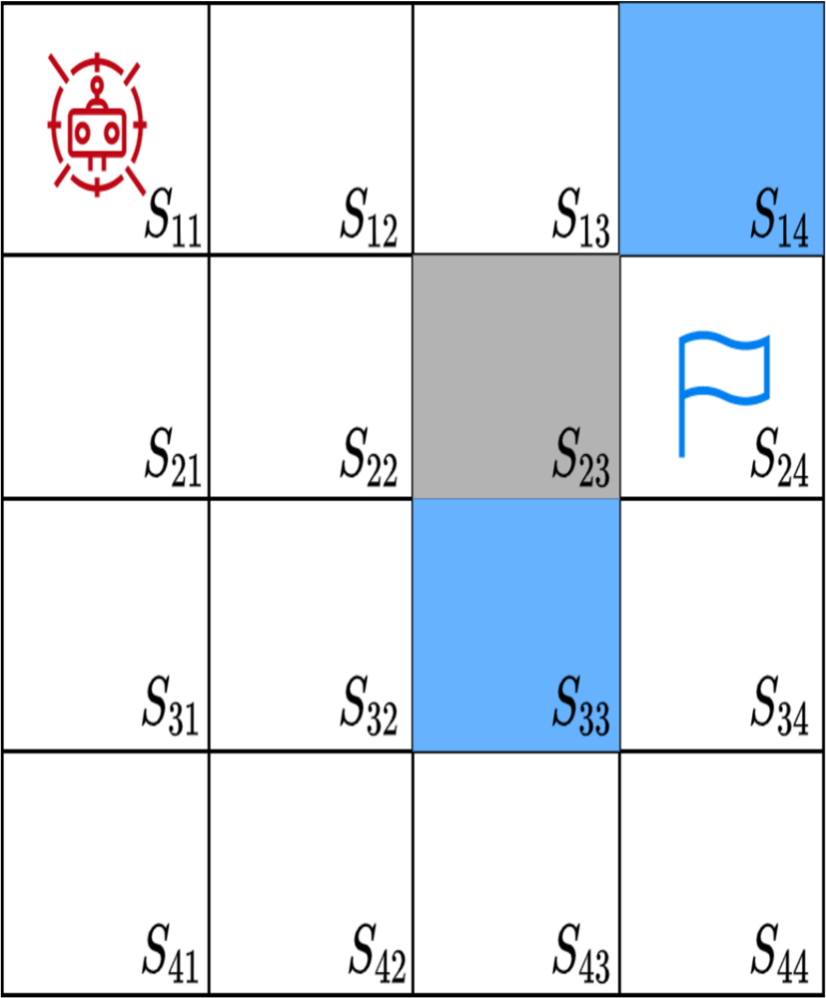
An example grid world with a 4 × 4 cell

The table represents a grid-world environment where an agent can perform actions and receive corresponding rewards. The environment states are labeled from $S_{11}$ to $S_{44}$, and the available actions include $A_{u}$, $A_{d}$, $A_{l}$, and $A_{r}$, which correspond to moving up, down, left, and right, respectively. For each state, the table displays the expected reward value for each possible action. If an action is not feasible from a particular state, it is marked as “N/A.” The “Total Reward” column indicates the cumulative reward for all possible actions from a given state.

The “Ways” column indicates the number of possible actions from each state. For instance, state $S_{11}$ allows two actions (moving right or down), while the state $S_{22}$ permits four actions (moving in any direction). This table helps assess and compare different policies (sequences of actions) for the agent within the grid-world environment, enabling the optimization of the agent’s behavior.

## Analysis and Interpretation of Experimental Outcomes

This section is divided into three subsections, each addressing a specific aspect of the experimental results and their analysis. The first subsection examines the planning process and variations in distance, Iteration, episode, and learning rate, as well as the count using the optimized reward for the proposed Q-Learning (QL) and Deep Q-Learning methods. Compares the accuracy of different techniques and examines the state and reward variations at various discount rates.

The second subsection explains the training and testing process in a continuously changing environment, using a prey capture scenario as a case study. In this study, all computational experiments were conducted on a standard laptop equipped with an 11th-generation Intel Core i5-1155G7 processor (2.50 GHz), a 64-bit operating system, and limited system memory. Given these hardware constraints, the experimental design adopted smaller grid environments (3 × 3 and 4 × 4) to ensure that the reinforcement learning simulations operated efficiently without exceeding available computational resources. Under this configuration, the CPU runtime remained within practical limits, recording approximately 25.05 s for the 3 × 3 grid and 36.56 s for the 4 × 4 grid, which is suitable for a conventional non-GPU machine.

However, Larger n × n environments lead to exponential growth in the state–action space, greater memory demand for Q-table or neural network storage, and longer training durations due to more complex exploration dynamics. Consequently, scaling the experiments to higher-resolution environments would necessitate more powerful hardware, particularly higher-end CPUs, GPUs, and larger system memory to maintain feasible runtime and training stability.

### Assessment of the Trajectory Path Planning Method

**Fig. 7:**
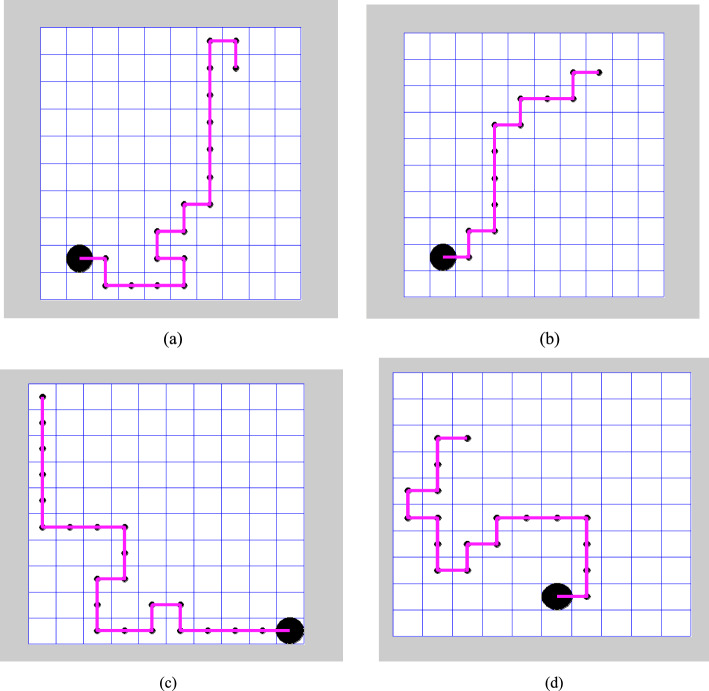
The structure of maps used for testing includes: **a** Map1 consists of both fixed start and goal points, with a combination of static and dynamic movement patterns. **b** Map 2 includes both fixed start and goal points, with a combination of static and dynamic movement patterns. **c** Map 3 features both random start and goal points, with dynamic movement patterns; and **d** Map 4 features both random start and goal points, with dynamic movement patterns

The proposed diagram for evaluating Q-Learning and Deep Q-Learning considers four different configurations, as shown in Fig. 7.

Figure 7 illustrates various positions of the agent, starting from the initial location at coordinates (2, 8). All paths in the grid are free of obstacles, allowing movement from the start to the goal position. The planned trajectory follows the shortest route to the target using left, right, up, and down movements, which may vary across episodes. The goal is located at the bottom-left corner, with coordinates (9, 2).

**Fig. 8:**
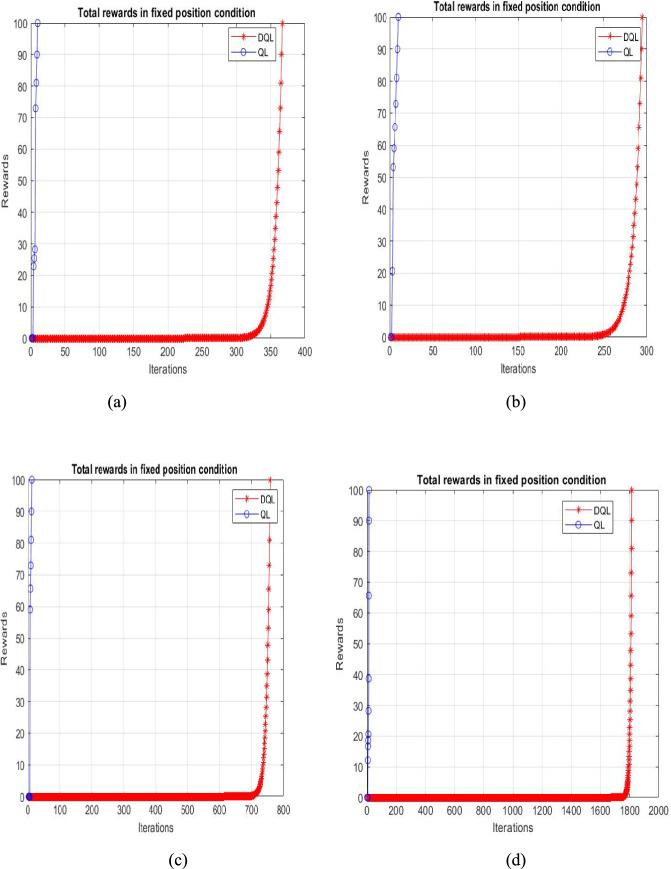
Graphical representation illustrating the relationship between cumulative rewards and training iterations for Q-learning and deep Q-learning algorithms

The second diagram, shown in Fig. 7b, modifies the original map by introducing randomly moving obstacles to assess how the agent responds to these changes. The third and fourth diagrams, in Fig. 7c and 7d, have random start and goal positions with moving elements. In these cases, the starting point is not (2, 8). Figure 8a–d show how episodes, rewards, and iterations are related to Q-learning in diagrams 1, 2, 3, and 4. The graphs demonstrate a consistent trend toward minimizing accumulated rewards. The similarity in accumulated reward values across the maps can be attributed to the uniform 10 × 10 grid size and comparable action sequence lengths for each map.

Graphical representation illustrating the relationship between cumulative rewards and training iterations for Q-Learning and Deep Q-Learning algorithms, highlighting the convergence behaviour and learning efficiency of each technique during the training process, as shown in Fig. 8.

**Fig. 9:**
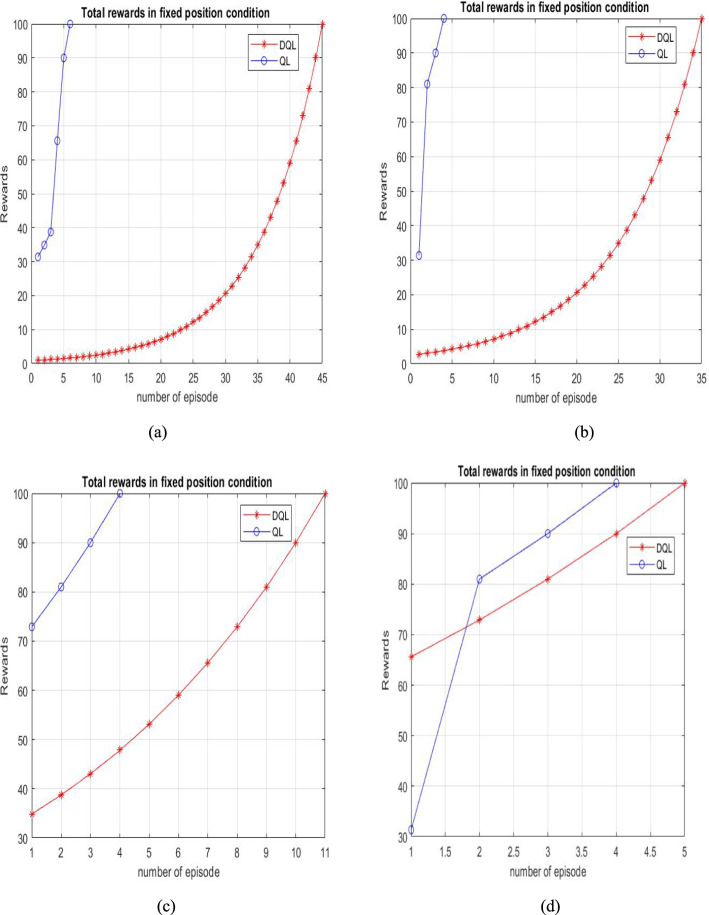
Graphical representation illustrating the relationship between cumulative rewards and training episodes for Q-learning and deep Q-learning algorithms

The performance of the proposed Deep Q-Learning algorithm is shown in terms of reward and total episodes for Maps 1, 2, 3, and 4 in Fig. 9a–d, respectively. These graphs indicate that convergence occurs around episode 100. The results demonstrate satisfactory performance, as the agent incurs minimal penalties throughout its trajectory. This is further supported by the near-zero values observed from episode 100 onwards, confirming the efficiency of the approach.

**Fig. 10:**
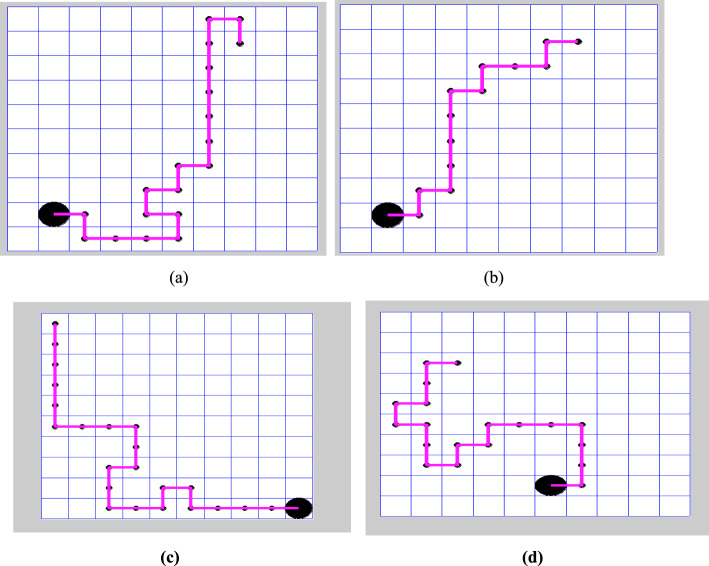
Graphical representation of the shortest trajectory, as determined by the proposed algorithm, is illustrated as follows: **a** the RL algorithm with fixed state achieved minimum distance over a 10 × 10 grid in 20 steps. **b** DQL method with a fixed state completed the trajectory over a 10 × 10 grid in 14 steps. **c** QL with random state navigated over a 10 × 10 grid in 23 steps. **d** The Deep Q-Learning algorithm with a random state successfully learned the trajectory over a 10 × 10 grid in 20 steps

The shortest trajectory is illustrated in Fig. 10, which utilizes the proposed algorithm, considering both fixed and random start and target positions.

**Fig. 11:**
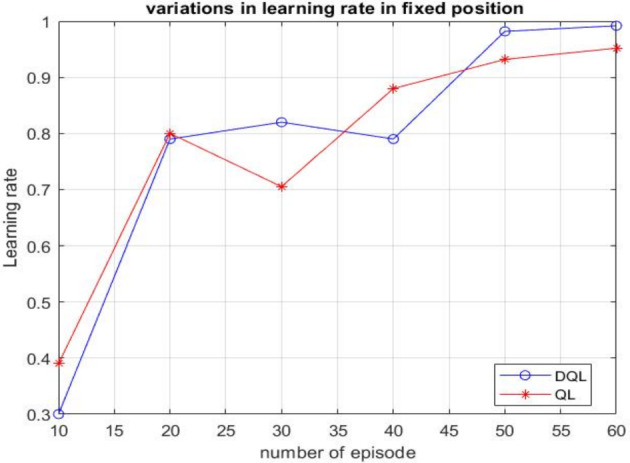
Graphical representation illustrating the relationship between cumulative rewards and training episodes for Q-learning and deep Q-learning algorithms

Graphical representation of the Deep Q-Learning (DQL) technique illustrating the relationship between the learning rate and the number of training episodes, highlighting how variations in learning rate influence convergence behavior and performance stability in Fig. 11.

In Fig. 11, the proposed trajectory planning model was analysed in comparison with Deep Q-learning, Temporal Difference learning, and RL-based Q-learning techniques. Findings demonstrate that Deep Q-learning achieves superior performance over the approach, particularly in terms of iteration count and the agent’s learning efficiency across various algorithms.

### Comparison of Accuracy Across Different Algorithms

The accuracy of various learning algorithms was evaluated, demonstrating that Deep Q-learning outperforms Q-learning (QL), Temporal Difference Learning (TDL), and DDQN in terms of learning rate efficiency relative to the number of iterations, as presented in Table 4.

**Table 4:** Accuracy comparison of various learning algorithms

Algorithms	Accuracy	Learning rate (L_1_)	Learning rate (L_2_)	Learning rate (L_3_)	Learning rate (L_4_)	Learning rate (L_5_)	Average (%)
Deep Q learning (DQL)	10	74.05	72.40	80.50	83.20	96.00	79.50
	20	97.05	77.53	67.81	85.30	97.51	82.10
	30	83.62	64.25	95.21	92.20	68.60	98.50
	40	99.50	83.04	95.40	84.50	96.50	88.90
	50	85.61	97.80	87.30	99.80	96.88	91.80
Q learning (QL)	10	76.06	75.80	71.08	85.50	95.09	78.80
	20	98.88	76.15	73.80	76.50	97.80	82.30
	30	85.56	67.56	94.40	96.80	68.50	79.40
	40	86.58	84.60	98.50	85.80	97.25	95.50
	50	85.58	97.80	82.50	98.80	95.80	91.80
Temporal difference learning (TDL)	10	93.70	75.45	71.81	76.54	98.65	83.54
	20	75.07	76.85	75.53	80.80	98.70	93.90
	30	77.63	69.40	95.87	94.55	63.53	78.02
	40	97.96	85.74	80.56	81.56	94.50	83.42
	50	99.40	88.40	90.40	83.20	96.40	85.70
Double deep Q network (DDQN)	10	80.10	80.60	75.60	84.30	93.40	80.10
	20	71.20	75.50	70.30	80.50	97.80	84.30
	30	84.32	67.60	98.60	97.50	63.50	78.30
	40	98.60	86.60	96.50	82.80	95.80	91.80
	50	84.05	97.80	82.40	99.50	99.80	91.80

Accuracy is assessed based on query evaluation using reinforcement learning with a neural network for each episode. Mathematical expressions are used to calculate the learning rate from Eq. (7), assess goal-tracking efficiency (EQ), and analyze overall training performance.

7 $$ {\mathrm{Eq}} = \left[ {1 - \frac{{{\text{Minimium }}\,{\mathrm{count}}\,{\text{ step}} \left( i \right) - {\mathrm{Total}}\,{\text{ count}}\,{\text{ step}} \left( {x1} \right)}}{{{\text{Total }}\,{\text{program }}\,{\mathrm{output}} \left( T \right)}} } \right]*100 $$

where T is the total number of states, the minimum count step is i, and x_1_ is the total count step.

**Fig. 12:**
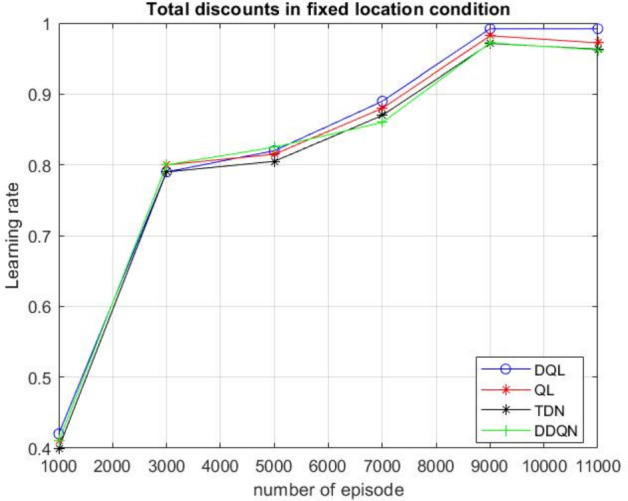
Comparison of return curves for four algorithms in a fixed-location environment, illustrating the learning performance over training episodes

In the experimental setup, four distinct algorithms were implemented for path planning within a controlled environment featuring fixed obstacles and goal positions. During the training phase, the agent progressively improved its navigation capability and was ultimately able to reach the target location without collisions across all four algorithms in the later stages of learning. The performance of each algorithm was evaluated by monitoring the cumulative return over training episodes, and the resulting return curves are illustrated in Fig. 12.

Figure 12 demonstrates that all four algorithms converge gradually toward the optimal reward value in the later stages of training. During the initial phase, the cumulative return of each algorithm increases rapidly as the agent begins to learn effective navigation strategies. Moderate fluctuations are observed in the mid-training phase due to ongoing exploration and policy adjustments. Overall, the DQL algorithm demonstrates comparatively higher stability throughout the training process.

The performance comparison of four algorithms in the fixed-position environment is shown in Table 5.

**Table 5:** Performance comparison of four algorithms in the fixed-position environment

Algorithm	DQL	QL	TDN	DDQN
The average learning rate	98.50	95.50	85.70	91.80
The average return values	6.12	6.51	6.62	6.66
The average rewards per step	0.098	0.097	0.093	0.94

According to Table 2, the DQL algorithm showed a faster learning rate compared to the QL, TDN, and DDQN algorithms. Specifically, the learning rates of QL and TDN were about 3 and 13 times lower, respectively, while DDQN was around 7 times lower than DQL. However, the QL and TDN algorithms achieved higher average return values than the DQL algorithm. Among all algorithms, the TDN algorithm had the lowest average rewards per step, whereas the DQL algorithm had the highest. Overall, DQL learned faster, but QL and TDN performed better in terms of return efficiency.

Based on the experimental evaluations presented in this paper, along with the data in Table 4, Q-learning evaluates a longer trajectory path of 23 units. In contrast, it determines a comparatively shorter route of 20 units, as shown in Fig. 9. However, we observed an increase in time complexity without achieving an optimal solution. In contrast, the proposed algorithm identifies the shortest decision path of 14 units in Fig. 9, demonstrating its efficiency. Deep Q-learning proves to be more effective in implementing trajectories involving repeated actions. One of its significant applications is in grid-world games, particularly in agent-based tasks such as prey capture. Furthermore, a comparative analysis of various reinforcement learning algorithms, as illustrated in Fig. 11, indicates that the introduced method outperforms others in terms of learning rate and the number of iterations.

According to Table 6, the optimized reward Deep Q-Learning model exhibits a significantly faster convergence pattern and reduced path-collision complexity compared to the QL, TDN, and DDQN algorithms.

**Table 6:** Performance comparison between baseline and optimized reward functions for Q-Learning and Deep Q-Learning

Measure	Baseline reward function	QL with optimized reward	Optimized reward (deep Q-learning)
Trajectory optimality	Small path length due to iterative learning	More optimal due to iterative learning	Most optimal with consistent shortest paths due to non-iterative
Computational cost	Low	Average	High due to neural network training
Decision path	Irregular trajectory paths	Regular or smooth path due to the state action pair	Highest smoothness due to continuous state action evaluation via a neural network
Convergence speed	Slow and unstable in large grids	Faster and stable convergence due to state action	Fastest and most stable convergence with the NN feature
Collision avoidance	High collision probability in dynamic scenarios	Reduced collisions with less reward	Lowest collision due to generalized and dynamic reward
Performance	Suitable for simple static grids	Ideal for simple static large grids	Suitable for dynamic large grids

### GUI-Based Framework for Prey Capture in Dynamic Environments: Game-Inspired Problem-Solving Approach

During the training process, the agent progressively learns to navigate the grid-world environment through repeated interactions and feedback from the reward function. In the initial episodes, the agent exhibits random exploration, frequently colliding with obstacles or taking inefficient routes toward the goal. As training progresses, the reinforcement learning policy gradually adapts, enabling the agent to identify optimal state–action mappings and minimize unnecessary movements.

**Fig. 13:**
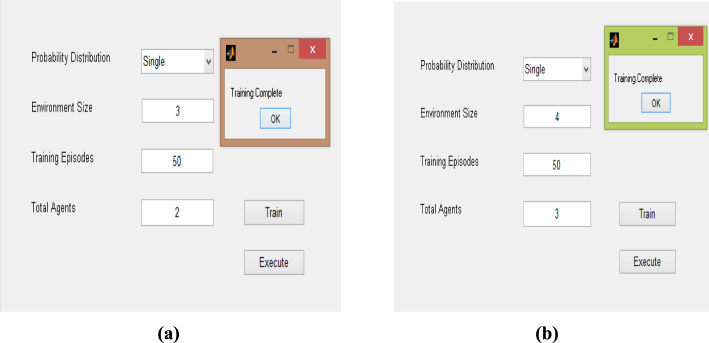
GUI The framework for prey capture using the DQL method shows: **a** multiple agents trained in a 3 × 3 environment for 50 episodes, showing learning and adaptation; **b** multiple agents trained in a 4 × 4 environment for 50 episodes, also showing learning improvement

For the 3 × 3 environment, the policy converges more rapidly due to the limited state space, resulting in smooth and consistent path selection. In contrast, the 4 × 4 environment requires a more extensive exploration phase, as the agent must evaluate a greater number of possible transitions and coordinate actions among multiple agents. Over successive episodes, the agents begin to cooperate implicitly, avoiding redundant paths and minimizing conflicts, which indicates effective policy evolution. Overall, the results shown in Fig. 13a and b demonstrate that the agent’s behaviour transitions from exploration to exploitation, leading to stable and goal-oriented navigation strategies across different environment scales.

**Fig. 14:**
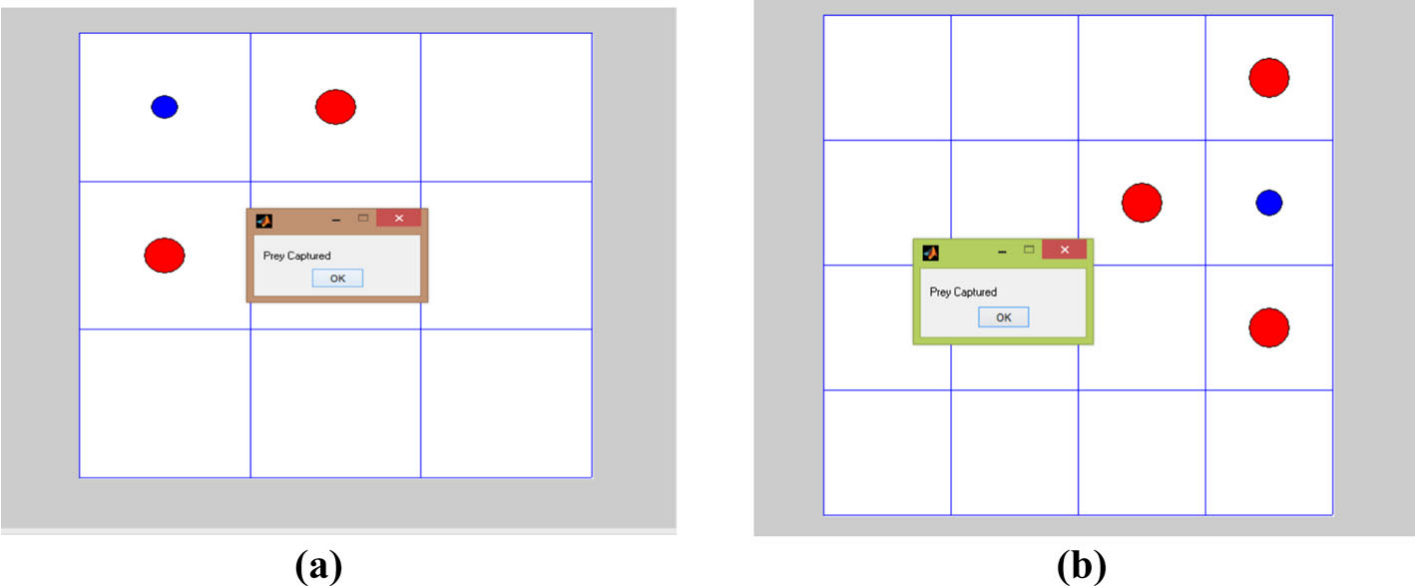
A GUI-based framework for prey capture in dynamic environments using the DQL algorithm is depicted: **a** testing of multiple agents in a 3 × 3 environment over 50 episodes, highlighting learning progression and adaptation; **b** testing of multiple agents in a 4 × 4 environment over 50 episodes, demonstrating similar learning improvements

During the testing phase, after the training process has achieved convergence, the agent effectively applies the learned policy to perform the prey capture task. Initially, the agent demonstrates a reactive and exploratory behaviour, adjusting its movements based on the prey’s position and environmental constraints. As the episode progresses, the agent utilizes its trained policy to predict and intercept the prey’s path more efficiently. Figure 14a and b illustrate how the agent’s actions evolve from basic pursuit strategies to coordinated and goal-directed movements, minimizing unnecessary exploration. The agent exhibits an improved ability to anticipate the prey’s motion, adapt its trajectory, and maintain optimal spacing to ensure successful capture. This indicates that the learned policy generalizes well beyond the training phase, enabling stable, adaptive, and intelligent behaviour in dynamic testing conditions.

#### Agent Training in Grid-Based Environments

In the context of multi-agent systems, it allows multiple agents to learn and navigate simultaneously within the same space. Each agent observes the environment, takes actions, and learns from its own experience, while also considering the behaviour of other agents. This setup is proper for real-world situations where many robots, drones, or autonomous vehicles share the same workspace. Multi-agent setups allow the framework to be extended to larger, more realistic applications such as warehouse robots, traffic navigation, and swarm robotics. With multiple agents, the system learns how to distribute movement so that paths do not overlap or block each other.

Multi-agent systems operating in grid-based environments (e.g., 3 × 3 or 4 × 4 grids) involve training agents to learn optimal strategies for achieving specific goals, such as capturing a “prey” agent, while navigating the environment. Here is a breakdown based on the observed data:

***Grid States and Environment:***

- **3 × 3 Grid**: A smaller environment where agents can explore up to 9 positions.
- **4 × 4 Grid**: A more complex space with 16 positions, increasing the state space and decision complexity.

***New States of Agents***

- These values (e.g., 8 6 for 3 × 3 and 8 3 6 for 4 × 4) represent the different unique states or positions the agents occupy or move to during training.
- The variation in states suggests that agents are exploring the grid effectively during training episodes, learning how to coordinate their movements.

***Prey State and Capture***

- The “Pray state” (likely meant as Prey state) refers to the position of the prey in the environment.
- “Possible prey states new = 0 0 0 0” implies that during these episodes, no new states for the prey were discovered—possibly because the prey was stationary or easily predictable.

***Capturing Time***

The capturing time reflects the efficiency of the agents in reaching and capturing the prey.

- 3 × 3 Grid: Capture time is approximately 25.05 s.
- 4 × 4 Grid: Capture time increases to around 36.56 s, indicating higher complexity.

To evaluate the scalability and computational efficiency of the proposed model, additional experiments were conducted with grid sizes of 12 × 12, 15 × 15, and 20 × 20. The time complexity of the generalized environment is O(n^2^), where n represents the grid dimension. The time complexity of the cell (*n x n*) is T(n) = O(n^2^). Where n is the number of cell states. Accordingly, the evaluated computational complexities for the respective grid sizes are 20,736, 50,625, and 160,000. As the grid dimension increases, both the state space and computational demand grow exponentially, resulting in significantly higher training time during the prey-capturing tasks. While the proposed algorithm maintains stable learning performance across different grid scales, larger environments inherently require more computational resources and longer convergence times due to their increased complexity. Smaller environments, such as 3 × 3 and 4 × 4 grids, reached convergence within fewer episodes (around 40–50), whereas larger grids required more training iterations due to increased state–action complexity. The runtime, CPU/GPU utilization, and the number of training episodes are necessary for convergence across different grid sizes (n × n cells).

The agents demonstrate learning progress by adapting to different grid sizes and effectively navigating to capture the prey. Increased capturing time in larger grids reflects the added complexity. No new prey positions suggest predictable prey behaviors, allowing agents to generalize their capture strategy efficiently.

**Fig. 15:**
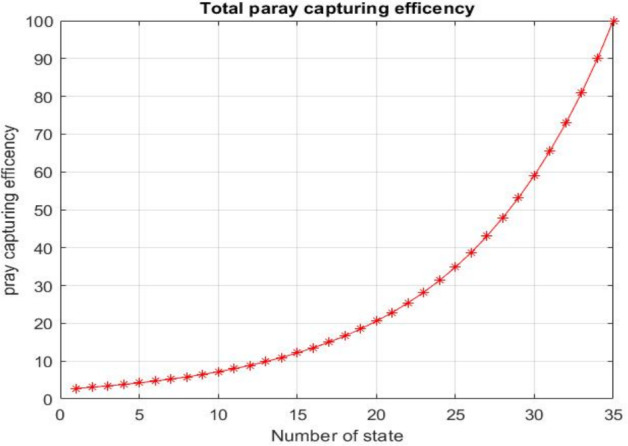
Comparative analysis of prey capture efficiency as a function of the number of states

Upon successful completion of all experimental trials, we compared prey capture efficiency, learning rate, and the number of states and episodes. The introduced approach achieves superior prey capture efficiency and learning rate, characterized by minimized delay rewards, reduced training time, and improved data retention—benefits applicable across game playing, robotics, and other domains. Analysis of prey capture efficiency as a function of the number of states, illustrating how increasing state complexity affects the agent’s decision-making accuracy and overall task performance, is presented in Fig. 15.

The results show that both Q-Learning (QL) and Deep Q-Learning (DQL) enhance agent performance by reducing the number of repetitions and episodes required to complete the task, even when the environment changes. These methods help shorten the agent’s path, cutting the number of steps from 20 to 14, which is a 30% to 70% reduction in both fixed and random setups.

Among the two, Deep Q-learning performs better by lowering the number of iterations and improving path planning. As shown in Fig. 10, DQL enables agents to complete tasks more efficiently in both Map 1 and Map 2.

The designed reward function supports better path planning by encouraging shorter routes and fewer steps. As shown in Fig. 12, Deep Q-learning outperforms both Q-learning and Temporal Difference Learning.

A practical example of prey capture in a dynamic environment is presented in Figs. 13 and 14, illustrating autonomous robots that adapt and learn to accomplish their objectives across different environments. These agents operate independently, autonomously developing diverse gameplay strategies through experience. Their efficiency underscores the successful application of reinforcement learning in artificial intelligence, particularly in robotics and related fields.

## Conclusions and Future Work

This study demonstrates that Q-Learning (QL) and Deep Q-Learning (DQL) enhance navigation by reducing the number of steps and episodes required to reach the goal, even in dynamic environments. The use of an optimized reward function helps shorten the path, leading to a 30% to 70% reduction in travel distance in both fixed and random setups.

Our results show that Deep Q-Learning is the most effective method for the prey capture game, with better capture efficiency. A comparison of DQL, Temporal Difference Learning (TDL), DDQN, and Q-Learning (QL) reveals the pros and cons of each method. One main drawback of Q-learning is that it often results in longer paths, which increases the time and number of steps needed to complete the task.

Future work can focus on utilizing multiple learning agents and advanced methods, such as transfer learning, to enhance the system’s capabilities. Testing these approaches on larger maps can help check how well they adapt and improve performance in changing environments.

## Data Availability

No datasets were generated or analysed during the current study.
